# Paranoid atmospheres: Psychiatric knowledge and delusional realities

**DOI:** 10.1186/1747-5341-4-14

**Published:** 2009-09-17

**Authors:** Jann E Schlimme

**Affiliations:** 1 Department of Psychiatry, Social Psychiatry and Psychotherapy, Hannover Medical School, Hanover, Germany; 2 Clinic for Psychiatry and Psychotherapy II, Paracelsus Medical University, Salzburg, Austria

## Abstract

In this paper I investigate the topic of paranoid atmospheres. This subject is especially of interest with respect to persons who are deluded, and also, I will demonstrate, sheds light upon the psychiatrist's "gaze" and knowledge of delusions. In my argument I will follow a path initially outlined by Karl Jaspers (1883-1969): modern psychiatric diagnosis of delusions is a diagnosis of form and not content. Jaspers' emphasis on the form of delusions enables psychiatrists to be self-critical about their professional knowledge and, consequently, prevent the development of dogmatic attitudes. In accord with Jaspers, my argument will focus on the basic structure of delusions and highlight the difference between delusional realities and non-delusional realities, a difference that follows from the possibility of self-criticism of one's own conscious and explicit convictions. I will demonstrate the importance of self-criticism with regard to paranoid atmospheres and also to psychiatric knowledge. In this manner, an understanding of delusions as lived experience will be developed, which argues that an escalation of the influence of delusional convictions, resulting in a profoundly paranoid atmosphere, is most problematic for the deluded person. To acknowledge this insight mirrors the need for a self-critique of psychiatric discourse, encourages an empathic and respectful relationship between professionals and deluded patients, and enables deluded persons to restrict their paranoid atmosphere. It is the main conclusion of my paper that a deluded person cannot do (with respect to his delusional convictions) what a psychiatrist must do (with respect to his psychiatric knowledge and his own existential convictions) in order to prevent a profoundly paranoid atmosphere in their relationship: be self-critical.

## Introduction

The reference point for psychiatry is the subjective experience of the person at hand. Prereflexive experience is undeniable and cannot be rejected, though a person can have doubts about the solidity or trueness of his experience. This prereflexive certainty of one's own experience is also true in paranoia and raises the question: how can a psychiatrist be so sure about the diagnosis of delusional convictions? In this paper I will first demonstrate that this question is still valid for us today. I will then discuss the relationship of delusional convictions and psychiatric knowledge in three dimensions: a) on the level of psychiatric knowledge including its historical background, which appears like a legacy to modern psychiatry; b) with regard to the psychiatrist as a person with self-critical abilities, c) with regard to the deluded person as an existence. Basically my argument will follow the same path in all three dimensions as outlined by Karl Jaspers (1883-1969): modern psychiatric diagnosis of delusions is a diagnosis of form and not content, which enables the psychiatrist to be self-critical about his professional knowledge and prevents a dogmatic conception of his knowledge. Following Jaspers my argument will focus on the basic structure of delusions and highlights the difference between delusional realities and non-delusional realities, the later are seen in the possibility for self-criticism of one's own conscious and explicit convictions. I will demonstrate the importance of this possibility for self-criticism in all three dimensions and will spell out its connections with what, in this paper, shall be called 'paranoid atmosphere'. Thereby I also develope an understanding of delusions as lived experience which integrates the first-person-perspective of the deluded person. It is the main conclusion of my paper that a deluded person cannot do (with regards to his delusional convictions) what a psychiatrist must do (with regards to his psychiatric knowledge and his existential convictions) in order to prevent a profoundly paranoid atmosphere in their relationship: be self-critical.

### I. Subjective experience, psychiatric knowledge and delusional convictions

In the following paragraph I will demonstrate that the question 'How can a psychiatrist be so sure about the diagnosis of delusional convictions?' is still of actual importance since it is in a structural way connected with the relationship between patient and psychiatrist, namely with the interrelation of the first-person-perspective of the patient and the psychiatric knowledge of the psychiatrist.

As I already said, the subjective experience of the person at hand is the starting point for psychiatry. Listening carefully and with empathy helps build a good relationship with the patient, which will activate resources for coping with symptoms. Furthermore, it offers the possibility to clarify the wishes, goals and values of the disturbed person, which gives the whole treatment perspective and direction. Yet, when trying to help someone who suffers from mental disorder, more is required than listening carefully and with empathy (though this is important). For a psychiatrist it is also necessary to look behind the report and narrative a person is giving in a situation of clinical exploration. This necessity becomes clear when considering the aims of psychiatric treatment from the patient's perspective. These aims are a) the relief of the patient's suffering, b) the patient's contentment with his way of living - which includes especially the interpersonal dimension - and c) the patient being in charge of his life again. The two last aims are contentious in the medical model, but not for a person-centered psychiatry when taking the first-person-perspective of the concerned person into account [1]. It is this patient's perspective that I will focus on in my paper.

In order to approach these aims it is not enough simply to feel with the patient empathically. If for example an elder patient is not drinking enough water in hot summer, it is possible that he becomes delirious and developes persecutory delusions. In this situation, it requires more than just feeling with him, but realizing that he is dehydrated and possibly also hyponatraemic. It is necessary to do something that has good evidence on its side to alter the subjective experience positively and to help the person to recover without harming him. In the given example this means starting a careful rehydratation therapy and controlling the sodium concentration in blood serum. For the psychiatrist it is therefore essential to understand the inner psychic structure and conditions predisposing, triggering and maintaining mental disturbances. In other words: psychiatrists are interested in the etiology of mental disturbances of the person at hand from a third-person-perspective as well as from a first-person perspective (subjective experience, inner reality, wishes and values). Psychopathological symptoms are of interest both as a subjective experience the person has to cope with and as a sign for a supposedly underlying pathological process.

A psychiatrist can't read minds, but armed with professional knowledge and training, he is increasingly able to detect signals people are unconsciously and non-verbally communicating and to become aware of aspects of a person that the person is trying to hide from himself. With regard to this twofold assessment of psychiatric exploration it is not surprising that a psychiatrist is somehow able to look deeply into another person's mind, maybe in certain aspects even "deeper" than the person himself. In the given example the psychiatrist's interpretation that the patient is suffering from dehydration is quite different from the patient's view, who is maybe convinced that the KGB is behind it all. Even though the psychiatrist gains no complete insight into another person's mind, it is no wonder that people get suspicious about the psychiatrist. Will an atmosphere of suspicion not provoke a more or less suspicious client? Will the suspicious client not hide things thereby provoking suspicion on the part of the psychiatrist?

With respect to the necessity of looking behind the subjective experience of the patient and interpreting his symptoms as signs of an underlying psychopathological process, we can state the thesis, that there is a latent paranoid atmosphere in the relationship of psychiatrist and patient. For psychiatry this is an important problem. What if the old man is in fact persecuted by an intelligence service and someone added a diuretic substance in his drink in order to provoke dehydration? Of course this sounds a little odd, but it shows the importance of the question: how can a psychiatrist be so sure about the distinction between delusional and non-delusional realities in his day-to-day business?

This problem accompanies psychiatry from the start, as I will show in this paper in the next step. In particular the anti-psychiatric movement criticised the positivistic attitude of psychiatric knowledge in the 60's and 70's of the last century. As Kotowicz argued, two main lines of argument were formulated: on the one hand, "madness" and other mental disturbances were viewed as a reflection of the "system" (e.g. the family, psychiatric institutions and treatments, society on the whole) and on the other hand it was observed, that our society and especially psychiatry did not perceive the creative and primary force of "madness" and its ancient truths ([2], p. 113). Surely delusions are only a special case of what can be called "madness", and there is strong evidence that the "trouble generator" of "madness" or schizophrenia is not primarily connected with delusions, but with self-disturbances [3,4]. Yet, at the basis of the anti-psychiatric arguments lies a deep scepticism regarding psychiatric knowledge. It is this scepticism that is connected with the question; How can psychiatrists be so sure about their distinctions between delusional and non-delusional realities?

This problem was not only addressed by the anti-psychiatric movement in a sceptical way, but already by Karl Jaspers in a more phenomenological one. His solution to the problem, the most favoured in the modern psychiatric discourse, is the insight that a) delusional realities are structured differently than non-delusional realities and b) the main reason for suffering is not the delusional convictions themselves but rather the escalation of these convictions and their taking over the entire reality of the person such that degrees of freedom are decreased. In the following I will argue that this insight, as can be found e.g. in the "Allgemeine Psychopathologie" (General Psychopathology) from Karl Jaspers [5] or in modern times in the writings of Michael Musalek [6], highlights and shows the need for self-criticism in psychiatric discourse. As already mentioned, it is my thesis that psychiatrists must be able to reconsider their professional decisions and knowledge with regards to the fact, that human beings are not able to discuss the question "Who am I?" exhaustively.

### II. The "psychiatric gaze" and the Enlightenment

In this section I will discuss the early history of psychiatry as an example and legacy of what can happen if psychiatrists do not remind themselves from time to time about the dangers that ensue when psychiatric knowledge becomes dogmatic to the degree that they are deemed by someone to be not falsifiable. It is my thesis that this danger is present if we lose the competence for radical self-critique, because a paranoid atmosphere always lurks in the background of psychiatry as already argued above on behalf of the difference between subjective experience and psychiatric knowledge.

Psychiatry developed around 1800 in different European countries and in the United States of America corresponding to a paradigmatic shift in medicine. As Nelly Tsouyopoulos in particular pointed out, the Brunonian system of medicine, a special vitalism developed by John Brown (1735-1788) and focusing on the excitability of the human body's tissue, turned the entire concept of disease upside down [7]. Until that time diseases were understood as natural entities ([8], p. 31-35). The basic theme of this former concept is the assumption that every disease has a somewhat typical feature more or less distorted by the individual body it resides in and that it has its own natural course. As a consequence, diseases are deemed to have their own and somewhat different processes than those going on in healthy persons. In this concept delusions were often understood as an obsession with an unfriendly spirit or a toxic miasma. The central aim of treatment is then to help the disease run its natural course, so that the disease can come to its preordained end and the body can be free of the disease again. In order to treat the disease correctly it is indeed necessary to make the correct diagnosis, which is complicated because the original disease is distorted by the individual body it has taken up with for residence.

In the new physiological paradigm things are understood totally differently: Diseases are now thought to use normal physiological processes in the human body - partially altering these due to the logics of the processes - and producing themselves using these processes. Symptoms of disease must then be understood as signs of the underlying process of the disease - the dehydration for example - and the job of the medical doctor consists of reading and deciphering the disease written in signs in body or mind of the individual. Another important change concerns the status of subjective well-being, that is distinguished from normal (psycho-)somatic functioning in Brunonianism. Subjective well-being is not simply a different quality of normal bodily functions and pathological functions does not necessarily lead to feeling ill [7]. In modern medicine this is one of the most important differentiations as can be seen especially in chronic diseases. As a consequence of this radical change in understanding the functioning of the human body the concept of diseases as natural entities taking up residence in the human body or mind lost scientific support. This is also true for psychiatric discourse, even though later e.g. Emil Kraepelin (1856-1926), one of the most important founders of psychiatry and especially creator of the distinction of schizophrenia and affective psychoses, assumed schizophrenia ('Dementia praecox') to be a natural entity [9]. These dramatic changes in medicine at the end of the 18th century mirrored the process of enlightenment in European culture at that time though the Brunonian system of medicine as well as closely related vitalism needed further enlightment in the future. With regards to the foundation of psychiatry, it is important to note that the enlightening of the inner world of human beings was a widespread cultural interest of enlightened individuals partially motivated by their hope to understand themselves better and was a broad social theme with political power in the awakening societies ([10], p. 397; [11], p. 128).

Many psychiatrists at that time agreed on the basic assumption that they could really see the animallike part of the human psyche in madness. They understood the "psychiatric gaze" in a way similar to the German doctor Johann Christian Reil (1759-1813), though often with slightly different attitudes or a different philosophical background ([12], p. 273). Johann Christian Reil (1759-1813), official medical doctor of the town in Halle and chair of medicine at the University of Halle from 1787, and from 1810 chair of medicine at the just founded University in Berlin, was the leading person regarding the invention and foundation of psychiatry in Germany, often compared with Philippe Pinel (1745-1826) ([13], p. 21). Reil did not only invent the word psychiatry ("Psychiaterie") in 1808, he also wrote an important textbook about psychic disturbances.

In his "Rhapsodies about the exertion of the psychic cure on disrupted mental conditions" (1803; Rhapsodieen über die Anwendung der psychischen Curmethode auf Geisteszerrüttungen), Reil reflected on psychic disturbances, especially madness, utilizing a mixture of vitalism and natural philosophy as influenced by Schelling [14]. With regard to madness, Reil's distinction of a "Seelenorgan" ("organ of the mind"), localized in the brain, and a "Gemeingefühl" (sensus communis), localized in all other parts of the nervous system, are of special interest. Whereas the "Seelenorgan" was the central point of the psyche, in which a rational synthesis of all other mental elements was possible and which could affect all other parts of the psyche, the "Gemeingefühl" was a compilation of the senses, affects, drives and imagination and more animal-like ([14], p. 32). The main cause of a mental disease was the imbalance of those two "organs": a missing rational synthesis of the "Seelenorgan" on the one hand and/or an intensified production of imaginations, incentives or affects and moods on the other hand, overburdening the abilities of the "Seelenorgan" of this person and therefore overflowing their consciousness, rendering it unevaluated and uncontrolled. As a consequence, the medical doctor assumed that he could directly see the inner reality - the products of the "Gemeingefühl" - of the mentally ill, because the synthetic function of the rational "Seelenorgan" no longer hid the inner reality from others via transformation. Furthermore it is assumed in this understanding that the "madman" is not able to criticise or evaluate his own experiences due to his impaired or overwhelmed "Seelenorgan", so that he cannot detect that his experiences are only imaginations produced by his "Gemeingefühl". A deep analogy can be found between the doctor's "clinical gaze" on the "madman" and the gaze of the "Seelenorgan" on its inner reality, because both gazes are assumed to see this inner reality straight and unaltered as it is produced by the "Gemeingefühl" ([12], p. 280).

In this assumption a paranoid atmosphere is hidden. If this assumption is true, the psychiatrist could decide whether a subjective experience, intention or behaviour is a sign of "madness" - since it would only be a product of the animal-like part of the psyche - or if it is a true subjective experience that can claim to be the experience of a healthy person. The consequences are immense. If our old man is in fact persecuted by the KGB and there is a hostile special agent trying to kill him with a diuretic substance causing dehydration and delirium, the psychiatrist's diagnosis of persecutory delusions would, with false confidence, clarify that the persecution by the KGB was unreal from the beginning and that the old man does not need to fear anything from them. That is of course great nonsense and the anti-psychiatric critique would then be right to say that psychiatric knowledge is positivistic and anti-psychiatric critique would then be right to say that psychiatric knowledge may itself be no more than dogmatic knowledge and even a form of delusion. And it would be dangerous, especially for our old man.

In this situation it is fruitful for psychiatrists to remind themselves from time to time about the danger that can occur when psychiatric knowledge becomes a non-falsifiable conviction. It is my thesis that this danger is in place if psychiatry loses the competence for radical self-critique, which can "bracket" the taken-for-granted distinction of a delusional and a non-delusional reality. Such a fruitful self-critique can be compared with a phenomenological approach, which can basically also be described as a "bracketing" of the natural experience meaning an artificial alienation of the "taken for granted" experience. This "bracketing" involves radically changing the point of view, so that one can discover through progressive phenomenological work how one's experience is inherently structured and given. The question is therefore how delusional experience is inherently structured, which leads to the formal definition of delusions as presented by Karl Jaspers.

### III. The psychiatric knowledge of delusion

In this section I will address the formal definition of delusional realities as presented by Karl Jaspers in a more detailed way and I will discuss the actual psychiatric knowledge of delusions with regard to this definition. Further I will demonstrate that this leads to an understanding of delusional realities that introduces the amending concept of a paranoid atmosphere, which is also of clinical importance as presented by Michael Musalek.

Though it sometimes seems as if there is no commonly accepted definition of delusion in modern psychiatry, most psychiatric textbooks favour an understanding of delusion according to Karl Jaspers. Jaspers rejected the idea that people with delusions have false beliefs about their situation, and argued that delusions represent a particular kind of knowledge ([5], p. 48). A person with persecutory delusions does not believe that he is persecuted; he *knows *that he is persecuted. Our old man is absolutely certain that the KGB is behind it all, and even the best argument will not convince him that this is not the case. For Jaspers, this differentiation of knowledge and belief was essential to understanding delusions.

Jaspers was not primarily interested in the theme or content of delusions, though he was of course interested in them secondarily because they were of outstanding importance for the deluded person. Rather he was interested in the structural change of knowledge that can be found in delusions. The knowledge of the deluded person, e.g. his certainty that he is being persecuted, shows an extraordinary high degree of conviction ('unvergleichlich hohe subjektive Gewissheit') ([5], p. 45) and is furthermore immune to any counter-arguments or alternative explanations. The conviction is therefore also incorrigible. This incorrigibility ('Unkorrigierbarkeit') is the central criterion for delusion in the Jasperian view ([5], p. 46). In accordance with the Jasperian understanding Berner pointed out that the two criteria 'extraordinary degree of conviction' and 'rejection of alternative explanations' ('incorrigibility') are obligatory for the diagnosis of delusion, but that the often impossible seeming content of the delusions is only an accessory criterion [15]. Though it is sensible to argue, that this incorrigible certainty of a conviction and the corresponding pre-reflexive interpretation of reality are only an extreme with regard to an uncertainty-certainty continuum, this does not question the basic Jasperian assumption regarding the structural change of knowledge in delusions ([6], p. 157).

Furthermore, most important for the persistence and maintenance of the delusions, the deluded person creates a paranoid atmosphere around him. With respect to maintaining the delusions and creating a paranoid atmosphere Musalek points out two factors ([6], p. 161). One factor is the self-dynamics of delusions happening on an interpersonal and prereflexive level. Basically this idea is very simple and maybe therefore frequently overlooked, since it means that delusional symptomatology itself creates via the non- and para-verbal interaction with others a paranoid atmosphere maintaining all delusional convictions: "People who are usually very open and friendly with others may react to the deluded patient with some reservation and resentment because of the patient's suspicious behaviour. This serves to reinforce the suspicion of the patient. In this manner a vicious circle may be established, amplifying and prolonging paranoid behaviours and ideas." ([6], p. 166)

The second factor is the meaning of delusions themselves, since they offer a 'safe' interpretation in an unsecure world, even though these ideas may need permanent defense in relationships with others regarding the label "lunatic". This intensifies the paranoid atmosphere, meaning that the "whole world" falls under the influence of the delusional convictions ('polarized delusions'). As a consequence this person cannot function normally in day-to-day life, because the delusional convictions are moving and motivating the person more and more and create a hermetic and static ('frozen', Musalek) paranoid atmosphere. Yet it is equally important to know that a person suffering from delusions in the Jasperian sense can still function normally if the delusions constitute merely a more or less parallel world he lives in ('delusions in juxtaposition'). This illustrates the well-known fact that persons with delusions seldom suffer from the delusions themselves but from the loss of freedom when the delusional interpretation starts to take over the first-person-perspective of the deluded person and creates a paranoid atmosphere [16]. This is especially true when non-delusional reality encroaches on a deluded person in a manner that contradicts his delusional convinctions. For example if our deluded person is certain that he does not need to pay taxes, the tax office will challenge this conviction in their claim that taxes must be paid. This not only creates a loss of degrees of freedom on the financial and/or forensic level, but can also provoke the creation and/or intensification of a paranoid atmosphere. While Jaspers three diagnostic criteria for delusions are non-interpersonal, the more important aspects with regard to the suffering and agony of the deluded person - the polarizing of the world and the maintenance of delusional convictions that influence the "whole world" (via the 'paranoid atmosphere') - are interpersonal [17].

Yet this paranoid atmosphere also offers narcissistic gratification for the deluded person. From an interpersonal standpoint the deluded person knows himself to be the most important person around, since the interest and behaviour of almost everybody is focusing on him: e.g. in persecutory delusions the persecuted person is of outstanding importance, since it is he being persecuted by the "whole world". This is even more pronounced in delusions of grandeur or in religious delusions. In this sense it can be said: every deluded person is a pop-star, but he is the only one who knows ([17], p. 142). Yet this paranoid atmosphere can also show a clearly negative attitude, e.g. when the person has the incorrigible conviction of blaming himself for everything going wrong in the world around him. Even in persecutory delusions this negative quality is present in the angst and dread of being persecuted anytime in any situation and from nearly anybody, because these things go together. Apparently it is a mixed blessing being the centre of the world, demonstrating beside the interpersonal quality also the power of paranoid atmospheres.

### IV. Paranoid atmospheres and alternative explanations - Lydia

In this section I will address a question which logically comes up with respect to the psychiatric knowledge described above: Does psychiatric knowledge of delusions truly solve the problem that arises with the potentially paranoid atmosphere of the psychiatrist's "gaze"? Drawing also on a clinical case I will demonstrate that psychiatric understanding of delusions in fact solves this problem, if and only if such understanding is open to alternative explanations - in one word: is corrigible or falsifiable - due to the inner logic of its basic assumptions. Only then it is not simply mirroring the paranoid atmosphere in the "psychiatric gaze" and replacing belief with a non-falsifiable and dogmatic (maybe sometimes even 'delusional') knowledge on its own. This answer apparently has at least two aspects: first it has to be clear what shall be open for alternative explanations and second corrigibility must be available to the psychiatrist in his day-to-day work. In the text that follows I will argue that the psychiatrist's knowledge of delusions is falsifiable on the concrete and patient-centered level, since it is a formal definition in the sense that every possible deluded person holds incorrigible convictions not open to alternative explanations. This means also that the Jasperian definition of delusions is itself falsifiable on the formal level, for example if anybody can think of a person who is deluded and does not hold incorrigible convictions which are not open to alternative explanations.

The first aspect of my answer addresses the problem that not delusions themselves but deluded persons are coming to seek our help. Jasperian diagnostic criteria for delusions do not address the question "How does the person feel?". In contrast with the assumption that deluded persons have false beliefs about their situations, Jasperian diagnostic criteria for delusions simply acknowledge the special structure of delusional realities. This means, that a person with persecutory delusions, let's call her Lydia, could in fact be being persecuted. Furthermore, the diagnosis "delusion" is not equivalent with profound knowledge of the deluded person, but offers only an in-depth comprehension of one aspect of this person.

Last but not least, Jasperian diagnostic criteria for delusions offer no certain knowledge about underlying processes of disease, even though some delusions show special characteristics due to the underlying process ([5], p. 45). Delusions in affective disorders, for example, typically correspond to the mood of the person. The delusional theme is often psychologically connected with the life-history of the deluded person [15]. Lydia, for example, developed delusional convictions in a two months interval when substituted for a colleague in an employment site where workers were routinely monitored and videotaped. Lydia had just started working again after pausing due to her two children and was employed as a secretary in the state Office of Criminal Investigation. She had problems coping with the tasks of her work, especially typing, and she made a lot of typing errors in her first weeks. Feeling ashamed about her bad performance, she secretly took the error-laden papers home and destroyed the sheets painstakingly by ripping them into small pieces. She developed the conviction that members of her office were persecuting her due to her taking the papers home and were even monitoring her private apartment. A few months later she was convinced that even the Secret Service was behind it. The delusional conviction didn't interfere with her work performance and she continued working without noteworthy problems for the next five years.

With regard to therapeutic aims it was important to develope an empathic and respectful rapport with Lydia, even if this is "only" the basis for concrete psychopharmacological treatment. In the case of Lydia this meant avoiding arguing about the truth or falsity of the delusions. Instead I as her psychiatrist argued that her degrees of freedom were minimized due to the escalation of the influence of those convictions. Nine years later Lydia is still convinced that she was monitored and persecuted in the first few months when acting for a colleague, but she is sure that some time after those months the real persecution ended and she became ill with persecutory delusions. Yet this conviction does not impair her day-to-day abilities and she lives her life in an unspectacular and completely normal way. In other words: it is not necessary to wipe out the delusional convictions once and for all in order to be therapeutically successful. Therapy is successful if the paranoid atmosphere of the delusions can be diminished, if the delusional conviction is brought in 'juxtaposition' or has become a simple private and personal delusional conviction again, which is meant here more in the sense of a hobby than an obsession.

Lydia is now, three years after the acute episode of mental illness, well aware that her conviction of being persecuted by the Secret Service after the first months was a delusional conviction and that the main reason for suffering and not governing her own life was the escalation of this conviction. This escalation led to a paranoid atmosphere through a long lasting and slow process of expansion, in which at the end for example a placard provoked the spontaneous idea ('Wahneinfall') that someone had just searched her apartment forcing her to take a cab to rush back and make the best of it. At this time she was almost constantly afraid that someone would search her apartment, so that she replaced the locks on the front door and all windows nearly weekly. All these activities created constant distress, anxiety and interference with day-to-day activities.

In that state of mind, Lydia needed to open herself to alternative explanations concerning her suffering, the loss of contentment with her way of living and her passivity and helplessness. As a deluded person she knew for certain that her persecutors were the reason for her great distress. As her psychiatrist I was convinced otherwise; that the underlying process of disease and especially the paranoid atmosphere were the main reasons for her suffering, but I was not incorrigible with this knowledge - after all persecuted persons can in fact be persecuted. I respected her delusional convictions as a possible interpretation of the situation at hand, though they had only extremely small evidence on their side. My main goal was not to correct Lydia's convictions, but to set limits to the paranoid atmosphere. A first step was building a relationship with her, in which we were able to develop alternative explanations e.g. for her tenseness in many situations which was associated with a formal disturbance of her thinking. Those alternative explanations gave her reasons to take a small dose of an antipsychotic medication which, of course, helped her to open her mind to even more alternative explanations of her actual situation. Yet this didn't alter her conviction of being persecuted in the beginning. Making room for alternative explanations implied setting limits to the paranoid atmopshere and vice versa. In the end the regained openness for alternative explanations helped Lydia get along with her life more contently and be in charge of her life again.

### V. Knowledge or Belief? The psychiatrist's need for self-critique

After having demonstrated the importance of self-critique with regard to psychiatric understanding of delusional realities in a person-centered and structural sense, I will in this section address the question: What enabled me to be content with "a smaller success" in treatment? I will argue that it is one's personal ability for self-criticism and that this personal ability is connected with what Jaspers called "existential convictions" or the ability to "believe" in a philosophical sense.

What enables the psychiatrist to respect the delusional convictions of the deluded person as a possible, though notwithstanding all the entailed 'safety' and narcissistic gratification, most unfavourable interpretation of the world? It is my thesis that this is possible with the aid of Jasperian criteria for delusions which free the psychiatrist from any need to discuss delusional convictions on the level of truth or falsity or the level of believe it or not. This becomes clearer by remembering the Jasperian distinction between knowledge and belief and his thesis that in delusions knowledge replaces belief; an important distinction for the psychiatrist in his day-to-day work. In the following I will spell out in a more detailed way the difference between knowledge as a falsifiable cognition and belief as a non-falsifiable cognition. Such a distinction helps psychiatrists avoid two problematic modes of interaction with deluded patients. On the one hand, being forced to lie to deluded patients for example in an affirmative way ("Yes, the Secret Service is behind it all.") or, on the other, being forced to become relativistic and deny for example that antipsychotic medications will help limit the paranoid atmosphere. Both consequences can be fatal in everyday work.

When trying to understand this more profoundly it is necessary to remember the main distinction between knowledge and belief. In the formation of knowledge as explicit knowledge lived experience is necessarily divided into a known object and a knowing subject. This splitting is preformed in intentionality, since consciousness is always *consciousness of *something and never consciousness on its own and without content. Yet, such splitting of intentional relatedness can never be absolute or perfect, as is the main thesis of Husserl's concept of intentionality, and can therefore only happen on a superficial level without really touching the underlying basis of intentionality even if the later is not consciously assured. In science this splitting takes place in the process of objectification with a scientific method, producing a falsifiable and method-dependent knowledge that has good evidence on its side and can be interpersonally agreed on. It is therefore an explicit and concrete knowledge. Often the constructive process of objectification as well as the underlying and necessary basis of intentionality are "forgotten", so that the known object or structure seems to have a life of its own in the world. This "forgetting" is not meant as a general critique but rather as a statement regarding the status of scientific knowledge. Remembering the underlying basis of intentionality and the constructive process of objectivation is the main source of scientific self-critique. Acknowledging this primary basis spells out why we are not all-mighty and completely autonomous constructors of our world and that there is a passive givenness on a phenomenal level [18]. This mirrors the well known fact that lived experience is always prior to our reflective consciousness, which means that we are already always given in a "pre-reflective self-awareness, including its immediate, implicit, nonobjectifying, and passive nature" ([19], p. 71).

The acknowledgement of this passivity of being given cannot be transferred into knowledge in an explicit and concretely measurable way, yet it can be integrated in life in the sense of a belief ([20], p. 99 and p. 282). In contrast to knowledge it is typical for the epistemological status of a believed content that it is not explicitly clear in every aspect. The believed content draws on special personal experiences, to which it refers at the same moment. These personal experiences are typical experiences of something incomparable, something indefinable in the end and can, for example, be possible in special situations via special techniques or practices. It can be argued that the epistemological status of a belief has nothing to do with transcendence or with this incomparable and indefinable self-affection. This is true. Yet it is important to notice that the epistemological status of a special content, which is believed and not known (due to impaired knowledge), is typically fashioned in a way that a last remaining uncertainty is spelled out as an explicit part of this special belief. In a delusional conviction exactly this last indefinability is lost. It is not *believed *to be the KGB, the deluded person *knows *that it is the KGB. It is this replacement of belief with knowledge that qualifies a delusional interpretation of the world as a safe island in the storms of uncertainty.

In his first edition of his "General Psychopathology" Jaspers comes to a similar conclusion. The psychopathologist should beware the borders of his possible scientific understanding: "This border is found if he is confronted with a single person, for he cannot dissolve this person completely in his psychological concepts. The more he brings his concepts to bear, characterizing and knowing as typical or regular, the more he gets to know that something incognizable is still hiding therein, something he can feel and suspect, but that he cannot grasp once and for all. For him as a psychopathologist it is enough to know about this infiniteness of every individual that cannot be exhausted; as a human being he may see more besides this; or, if others are seeing this incomparable more, he should at least not be meddling in it with his psychopathology." ([5], p. 1, translation mine) Jaspers names three typical ways for a psychopathologist to overstep the borders of his scientific conceptions: a) the 'somatic preconception' (das somatische Vorurteil) especially in the sense of a 'brain mythology' (Hirnmythologie), b) the 'philosophical preconception' (das philosophische Vorurteil) especially in the sense of a non-experienced speculative conceptualization and c1) the 'escalation of correct assumptions' (Übertreibung richtiger Anschauungen) for example leading to the conviction that people believing in a divine genesis are completely uneducated or c2) the 'rendering absolute of special standpoints' (Verabsolutierung einzelner Gesichtspunkte) in the sense of being a stickler for principles or in the sense of overgeneralizing, meaning for example being convinced that elephants have tusks and that therefore female Indian elephants can be not real elephants ([5], p. 9). These ways are all equal with regard to replacing belief with knowledge. In this replacing they become incorrigible, non-falsifiable and dogmatic and, in a certain sense, even comparable with delusional convictions.

Even more pointed is Jaspers critique regarding this problem in his later works after developing his existential philosophy. The most important idea regarding his existential philosophy is his rejection of a possible absolute conviction on the level of conscious and method-dependent knowledge and his absolute conviction that absolute convictions can only have the character of existential beliefs ([21], II p. 232 and III p. 137; [22], p. 44; [23], p. 16). Contents of belief "remain in the abeyance of being not-known [...] They are addressed and used too quickly as a knowledge and thereby lose their sense." ([23], p. 33, translation mine) Life is a good example for this. No one really knows what life is though we are all absolutely certain of being alive. If someone claims to know exactly and to the point what life itself is, it can be suspected that this person doesn't understand much, since life is always more than any conception of it.

The believed content is only meaningful for and is bound to the believing person, overcomes the subject-object-splitting and can be complete nonsense for another person. Therefore believed contents are existential convictions ([23], p. 16). Jaspers understood the content of existential convictions as "Chiffre", which is the immanent reality of transcendence. The "Chiffre" as a symbol has a double meaning: it symbolizes something infinitely more than the symbol itself and is on the other hand still just an immanent symbol ([21], III p. 141). A "Chiffre" is not necessarily a sign, it can be a natural formation like Ayer's Rock or a book like the bible. Life for example is symbolized in many ways and the most famous sign for life is the old Egyptian hieroglyph "ankh", the coptic cross. The coptic cross is not life itself, but as a symbol for the true phenomenon of life it refers to. Following Jaspers this duality shall not be understood as such: "The *Chiffre *is the being, bringing transcendence into presence, without the need for transcendence becoming a being as an object or for existence becoming a being as a subject." ([23], III p. 137, translation mine) The question remains: does this not lead to the imagination of a transcendence "behind" the "Chiffre", for which the "Chiffre" is just the clue and the symbol? If this would really be true, then an important question comes up, since then existence in the Jasperian sense would mean something "behind", "beneath" or "beside" the concrete person. Then we would have to face the problem of extreme relativity in the sense already mentioned above. As I will argue in the following part, Jaspers was well aware of this danger of extreme subjectivism. Even though existence in the Jasperian sense is principally an option for every human being, it is necessary to acknowledge that one's concrete beliefs are at least partially one's own product. With psychiatric knowledge in the background it is easy to see that exactly this is problematic for a deluded person. Yet I will argue in the last section that a deluded person can be understood as existence in the Jasperian sense if the person's perspective is not dominated by the delusional convictions respectively if the paranoid atmosphere does not encompass the "whole world".

### VI. Future prospects: being deluded and existence

In this section I will discuss whether a deluded person can be understood as an existence in the sense of Jaspers. I will demonstrate that especially the escalation of delusional convictions - in the sense of creating a profound paranoid atmosphere - prevents existing in the Jasperian sense and that this corresponds to clinical experience, as demonstrated in a second clinical case, that delusional 'polarization' of the "whole world" is the main reason for suffering.

Existence in the Jasperian sense is principally an option for every human being. It is generated in border situations: "The truth of eudaimonia evolves from the bottom of failure." ([21], II p. 232, translation mine). The performance of border situations means that the person realizes that the limits of his being cannot be changed but can be adopted as fundamental structure of his own existence. One of the border situations Jaspers names, besides death, affliction/suffering, guilt and historicity, is struggling (fighting, German: 'Kampf'). "Inevitably I have to co-create struggling and being involved in it as a border situation, I can get aware of myself existentially and adopt this border situation." ([21], II p. 233, translation mine) In the long run a fight is a matter of life and death, though the inescapable need to struggle does not mean chaos or violence. Instead it asks every person to acknowledge and adopt it as a situation inescapably mine. Even in love struggle is a inherent aspect of existence: "Love is no calculable possession. I have to struggle with myself and the existence of the loved one, without violence, but challenged and challenging. [...] Love as a concrete event in time is always incomplete and imperfect." ([21], II p. 244, translation mine).

Obviously existing in the Jasperian sense implies accepting and adopting this contradictory structure of being (antinomische Struktur des Daseins') ([21], II p. 250). Jaspers points out that existence affords a clear distinction from simple being, since existence implies being aware of one's own limits ([21], II p. 284). This can become clear at the "wailing wall of the existence" [24], though the mere insight into this border situation as an inescapable part of one's own existence does not necessarily mean that a person has the power and the ability to overcome his wailing wall and to adopt the limits of this situation on a new level of his existence. Jaspers was well aware of this difficulty: "I am existence only in unity with the knowledge of transcendence as the force, through which I myself am [...] Without existence the sense of transcendence would perish" ([22], p. 44, translation mine) Furthermore both existence and transcendence cannot be defined with concrete criteria, because then "the original thought of encompassment would be lost" ([22], p. 54, translation mine).

But is it enough to be aware of one's deepest indefinability in order to overcome one's wailing wall and to endure in life as existence? Robert, a seventy year old man and a maverick all his life, was very well aware that he as a person and life as a phenomenon could not be explained completely. Yet he was truly suffering from not being able to sleep at home anymore due to his conviction that he was being persecuted by an unknown organisation and in danger when sleeping in his bed. For over twenty years he often slept in his car parking somewhere in the vicinity of his home or even far away on a parking place near a autobahn. His reason for seeking help was the simple fact that this way of living had become too exhausting for him in his old age. Obviously it is not enough to know about this deepest indefinability of oneself to prevent delusional convictions.

On the other hand existence affords a rational attitude ([22], p. 48). "Existence is in need of reason. It becomes unsettled and aware of transcendence's claim in the light of reason, gets into its true movement via the prick of reasoning. Without reason existence is idle, sleeping, as if it isn't there." ([22], p. 49, translation mine) Yet a rational attitude is not enough in order to become an existence in Jaspers' sense. Jaspers points out that an "existenceless reason" dissolves into an "anything goes"-attitude because then the person has the illusion that one needs not commit oneself to life ([22], p. 50). The rational attitude is therefore a necessary but not sufficient condition for existing. Existence is no grand intellectual event but rather depends on nature and one's own being and can best be understood as the relying of existence and reason upon each other, illuminated in Jaspers' sentence: "Our essence is Being-on-the-way." ([25], p. 109, translation mine) For Jaspers existence produces beyond the simple reality of being another, "higher" reality, which can give the being distinction and limits, but cannot generate it and remains dependent and is well aware of this relying on each other ([22], p. 56).

If existence in the Jasperian sense relies on existential convictions as personal experiences, they are fundamentally not knowledge, but related to a preobjectifying experience, an experience of suspendend subject-object-splitting. Are not deluded convictions the extreme opposite of these existential convictions? What then about deluded persons, what about Robert and Lydia and their possibility of reaching the level of existence in the Jasperian sense? Jaspers answered, to my knowledge, only indirectly: "He who gives himself up submitting to apotheosised objectivities, loses himself as possible existence and therefore the probability of a primary and evident recognition of his transcendence. He only gains secure footholds, structure of being and the edifying natures of illusionary transcendence." ([21], II p. 145, translation mine) This theme can be found in a great variety in Jaspers works as already showed above (see also [26], p. 460 and p. 682 and p. 726). A person with dogmatic convictions sets the content of his convictions as absolute and they gain the status of knowledge. As a consequence this person "stands under dominating conceptions and thoughts, lives in a more or less conscious dogma and in corresponding preconceptions." ([26], p. 726, translation mine) Though Jaspers does not mention the comparableness of delusional convictions and dogmatic convictions, and there can be doubts whether this is really a meaningful comparison due to the intersubjective characteristic normally given in dogmatic convictions and only seldom present in delusional convictions (with the exception of a "folie a deux"), it is obvious that for Jaspers a deluded person is not existing in his sense. The deluded person would need to acknowledge that his delusional convictions are at least partially his own product and that he co-created them. With psychiatric knowledge and Jasperian criteria for delusions in the background we must confess that exactly this is not possible for the deluded person. In one word: this impossibility is exactly his problem.

Still Jaspers' concept of existence resists all intentions to break it down into special psychic functions, even though existence in the Jasperian sense relies on psychic functions. The deluded person, for example, has impaired mental functions and can therefore not criticize his delusional convictions. Yet this impairment is a very special one. The deluded person is not completely unable to criticize things rationally, but the critic is in service of the delusional convictions. The impairment means exactly this impossibility to criticize one's own delusional convictions and it is this impossibility that qualifies them as delusional convictions leading possibly to a paranoid atmosphere. It is not the delusional convictions that are the main problem for the deluded person, but the 'polarization' and the paranoid atmosphere. Robert could never comprehend that his loneliness was an important factor for maintaining his ongoing experiences of persecution, even though the persecution was less intense from that time on when he had weekly contacts with a cognitive training group and me as his psychiatrist, in which we took a short stroll together through a park talking. In that time he could normally sleep at home again, the paranoid atmosphere was limited to bearable dimensions. He still continued 'his way of living' - namely sleeping in the car - from time to time, though he never said he did this of his own volition. Yet personally I suspected that he did it sometimes also because he liked it. Robert as a deluded person with delusions in 'juxtaposition' can well be considered an existence in the Jasperian sense. His way of living became difficult due to the decline of his vitality in old age and the delusional convictions started to dominate his day-to-day life more and more. Obviously it is the 'polarization' of the "whole world", the subsumption of the whole lived experience and all that is given under the delusional conviction and the creation of an encompassing paranoid atmosphere, that hinders the adoption of one's own "wailing wall of existence" in a different way than defined by the delusions. It is an escalated paranoid atmosphere that prevents existence. The central question for a deluded person is therefore, if the 'polarization' can be stopped and if the paranoid atmosphere can be reduced.

## Conclusion

Paranoid atmospheres are of fundamental importance for psychiatry. A paranoid atmosphere is not only important with regard to deluded persons, but also with respect to the "psychiatric gaze" and the question whether a person can be understood as an existence in the sense of Jaspers. As could be shown a paranoid atmosphere is also hidden in the structure of the "psychiatric gaze", since it is necessary as a psychiatrist to look behind the report and narrative of the patient at hand when trying to understand the structure and etiology of his disease. This suspicion mirrors the changes of the "clinical gaze" around 1800 as well as the ongoing enlightenment at that time, even though this paranoid atmosphere basically founding the "psychiatric gaze" needs enlightment itself. Psychiatric knowledge is method-dependent and therefore in the need of self-criticism. It is important for psychiatrists to realize that the patient in front of them is more than psychiatric knowledge can describe. This insight implies to further realize that a last indefinability of a person as a human being will always remain. Though this last indefinability cannot be formulated as knowledge and has a status of a belief, it is this basis that allows the psychiatrist to acknowledge the deluded person's interpretation of the world as truly his interpretation. This is mirrored in psychiatric knowledge, that the structure of delusional convictions can best be described by the replacement of belief with knowledge and that the suffering of the deluded person and this loss of degrees of freedom is bound to the escalation and self-dynamics of delusions best described as a paranoid atmosphere.

I tried to show that a deluded person can be understood as an existence in the sense of Jaspers if the delusional convictions don't create a paranoid atmosphere. For existing in the sense of Jaspers it is necessary to preserve existential beliefs, whereas delusions offer 'safety' when a person cannot believe on an existential level anymore. In delusions, indefinability, transcendence and encompassment are driven into a subject-object split, fixed as knowledge and appear therefore "displaced". Yet they are in a deeper sense only "displaced", when such displacement hinders existing in the sense of Jaspers. This occurs when delusional convictions polarize the "whole world", since, consequent to the creation of a profoundly paranoid atmosphere, the person no longer acknowledges that the question of truth cannot be answered exhaustively. Deep scepticism regarding psychiatric knowledge shows that the real problem is not the delusional convictions in themselves but rather the escalation of the influence of delusional convictions. With regard to this insight it can be said that therapy is successful when the paranoid atmosphere of the delusions can be diminished, and the delusional conviction is brought in 'juxtaposition' or has become a simple private delusional conviction again. It is the acknowledgement that the suffering or enjoyment of a person, the contentment with one's way of living, and the question of whether a person is in charge of one's life are necessarily answered personally, in spite of all possible third-person answers, that opens these central aims of life to alternative explanations. Consequently, such questions can never be answered in absolute terms - and they are not necessarily already answered with the delusional convictions. Openness enables the psychiatrist to have an empathic and respectful relationship with his deluded patient, thereby mirroring the need for self-criticism of the psychiatric discourse, and it enables the deluded person to live a life as an existence - at least as long as his paranoid atmosphere is not polarizing and encompassing the "whole world". This can be supported through an empathic and respectful relationship, in which one (the psychiatrist) is mindful of the epistemological status of knowledge and belief, so that one's own ability (the psychiatrist's ability) for self-criticism prevents the creation of a profound paranoid atmosphere in the patient's experience.

## Competing interests

The authors declare that they have no competing interests.

## Authors' informations

Jann E. Schlimme is a senior lecturer for psychiatry and psychotherapy at Hannover Medical School (MHH, Germany) and leads the section "Phenomenological Psychiatry, Psychiatric Anthropology and History of Psychiatry" integrated in the Department for Psychiatry, Socialpsychiatry and Psychotherapy (head: Stefan Bleich; former head: Hinderk M. Emrich) at the Center for Mental Health of the MHH. He was also a senior registrar in this Department until 08-2009. From 09-2009 he is working as a senior registrar at the Clinic for Psychiatry and Psychotherapy II (head: Friedrich M. Wurst), University Clinic of the Paracelsus Medical University Salzburg, Austria. Jann E Schlimme holds an MD from Hannover Medical School since 1998 and received his licence to lecture in 2007. He also studied Sociology, Social Psychology and Philosophy at Leibniz-University Hannover (Master of Arts, 2004) and is actually working on his dissertation thesis in philosophy regarding the impact of a phenomenological enquiry of suicidality for mental health services.
